# A Case of Nasal Glial Heterotopia in an Adult

**DOI:** 10.1155/2014/354672

**Published:** 2014-02-20

**Authors:** Akira Hagiwara, Noriko Nagai, Yasuo Ogawa, Mamoru Suzuki

**Affiliations:** ^1^Department of Otolaryngology, Tokyo Medical University, 6-7-1 Nishishinjuku, Shinjuku-ku, Tokyo 160-0023, Japan; ^2^Department of Otolaryngology, Kohsei Chuo General Hospital, 1-11-7 Mita, Meguro-ku, Tokyo 153-0062, Japan

## Abstract

We report a rare case of nasal glial heterotopia in an adult. After the surgery, frontal lobe cerebral hemorrhage developed. A 58-year-old man had unilateral nasal obstruction that progressed for one year. He had been treated for hypertension, chronic heart failure, and cerebral infarction with aspirin and warfarin. A computed tomography scan showed that the tumor occupied the right nasal cavity and the sinuses with small defect in the cribriform plate. The tumor was removed totally with endoscopy. After the operation, the patient developed convulsions and frontal lobe cerebral hemorrhage. The hemorrhage site was located near a defect in the cribriform plate. Nasal glial heterotopia is a rare developmental abnormality, particularly rare in adult. Only few cases were reported. We could not find any report of adult nasal glial heterotopias that developed cerebral hemorrhage as a complication of the surgery.

## 1. Introduction

Nasal glial heterotopia, also known as nasal glioma, represents a collection of normal glial tissue in an abnormal location; the tissue is isolated from the nervous system without intracranial connection [1, 2]. Nasal glial heterotopia is a rare congenital lesion that is thought to be the result of abnormal embryonic development. It is frequently diagnosed in newborns or infants, although it is rarely found in adults. Histologically, these tumors are composed of astrocytes and neuroglial cells interlaced with fibrovascular connective tissue that is covered by the epithelium or respiratory mucosa [3].

Nasal glial heterotopias have no communication with the central nervous system. However, from CT findings, 15%–20% of patients have a defect in the cribriform plate [4, 5].

We report a case of nasal glial heterotopia in an adult man. The tumor was removed endoscopically. After the surgery, frontal lobe cerebral hemorrhage developed. We found that the hemorrhage site was located near a defect in the cribriform plate.

## 2. Case Presentation

**Figure 1:**
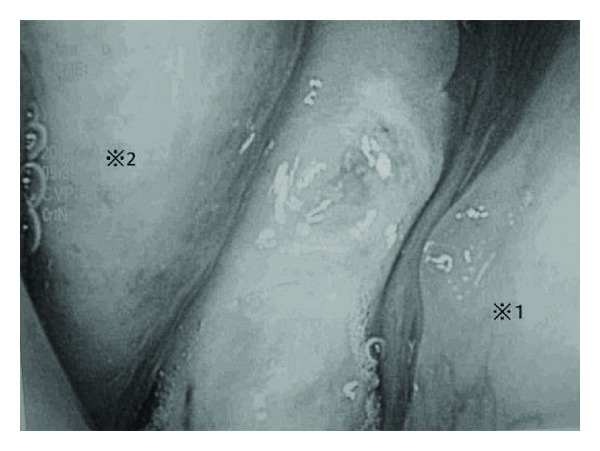
Endoscopic findings of intranasal lesion *※*1: Septum *※*2: Middle turbinate.

**Figure 2:**
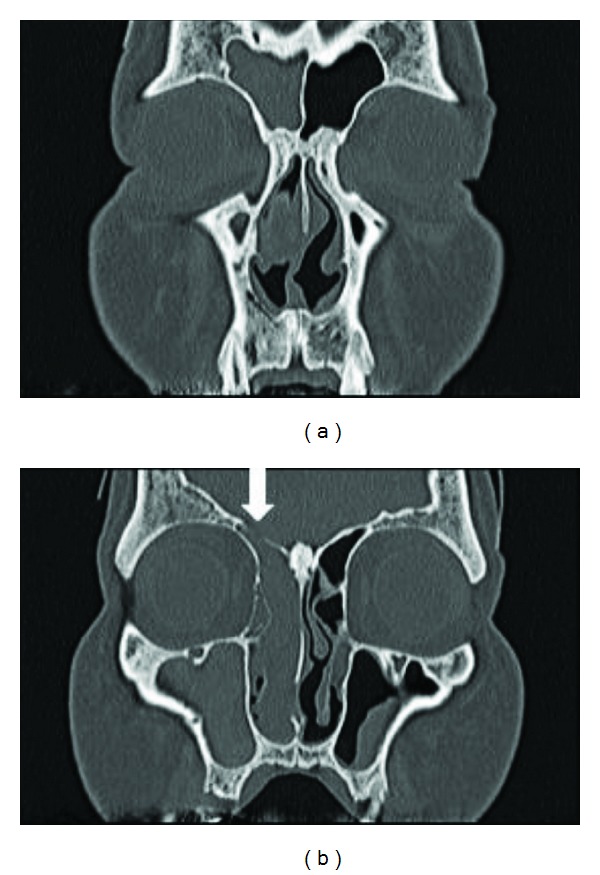
CT showed an isodense mass which occupied the right nasal cavity, maxillary, ethmoid, and frontal sinuses, with no erosion of the bony walls. ↓: A small defect in the cribriform plate.

**Figure 3:**
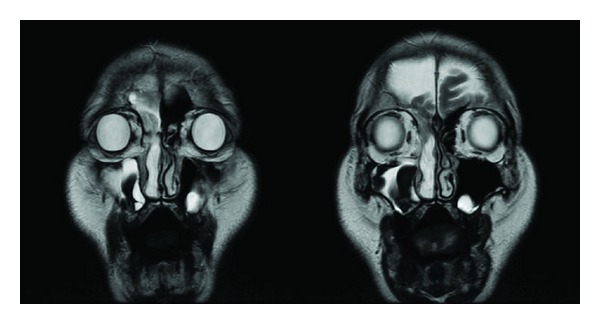
MRI showed that the tumor had high signal intensity surrounded by an isointense area on the T2-weighted image. No connection was observed with the meninges or cerebral tissue.

A 58-year-old man was referred to our hospital for evaluation of a unilateral nasal obstruction. His symptoms had progressed for one year before the consultation. He had no rhinorrhea or nasal bleeding. He had been treated for hypertension, chronic heart failure, and 2 incidences of cerebral infarction (the latter with aspirin and warfarin). Endoscopic examination revealed a soft, white, and polypoid mass that occluded the right nasal cavity (Figure 1). A computed tomography (CT) scan of the facial bones showed an isodense shadow, which occupied the right nasal cavity, maxillary, anterior ethmoid, and frontal sinuses, with no erosion of the bony walls (Figure 2). There was a small defect in the cribriform plate. Magnetic resonance imaging (MRI) showed that the tumor had high signal intensity surrounded by an isointense area on the T2-weighted image (Figure 3). No connection with the meninges or cerebral tissue was observed.

Total endoscopic resection of the tumor was performed under general anesthesia. The tumor was found to arise from the frontal sinus and was occupied the anterior ethmoid sinus and nasal cavity. The maxillary sinus was filled with the pus by secondary inflammation from the tumor. The tumor was removed under endoscopic visualization at 0 and 70 degrees of view. No cerebrospinal fluid (CSF) leakage was found during or after the operation.

Three hours after the operation, the patient developed convulsions. The CT showed frontal lobe cerebral hemorrhage. He was treated in the intensive care unit with an anticonvulsant sedative. One day after the convulsion, he became fully conscious, without signs of dyskinesia.

**Figure 4:**
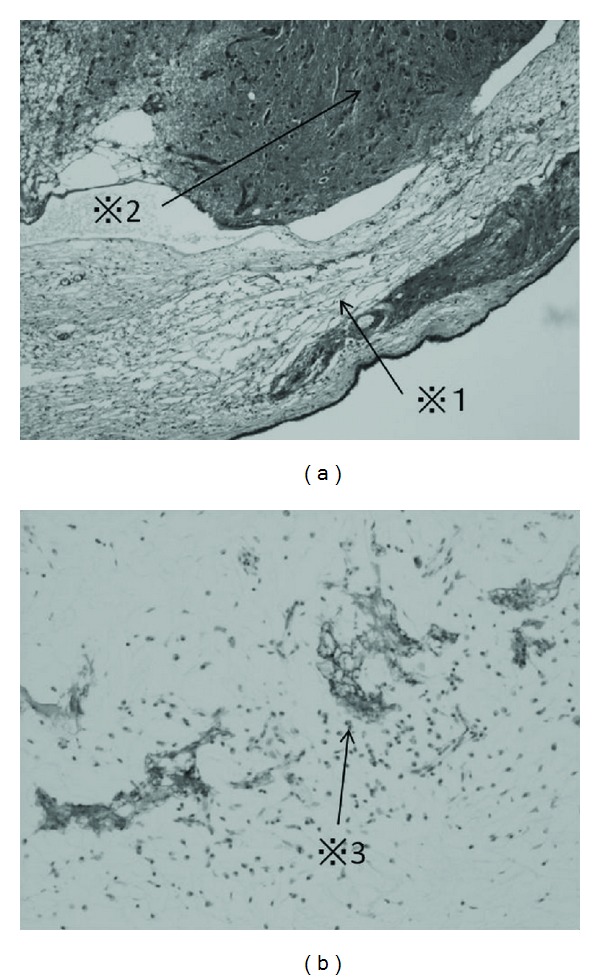
(a): glial cells surrounded by the respiratory epithelium with vessels and connective tissue. *※*1: respiratory mucosa, *※*2: glial cells (b): intense staining of glial elements for glial fibrillary acidic protein (GFAP). *※*3: stained cells.

Histopathologically, the tumor consisted of neuroglial cells, neuroglial fibers, and vascularized connective tissue. The tumor was surrounded by normal respiratory mucosa. The neuroglial cells were stained positive for glial fibrillary acidic protein (GFAP) (Figure 4).

For 2 years after surgery, the patient has been free from nasal symptoms, signs of recurrence, and dyskinesia.

## 3. Discussion

Nasal glial heterotopia (also known as nasal glioma) is a rare developmental abnormality typically seen at birth or in early childhood. Nasal glial heterotopia develops from normal glial tissue, but is located in an abnormal location, and is isolated from the nervous system without intracranial connection. It is thought to be the result of abnormal embryonic development. Approximately 250 cases have been reported [6], but only a few adult cases are known [3, 7–10].

Regarding the location, 60% of the masses were located extranasally, 30% were intranasal, and 10% were both [11–13]. In the intranasal type, most of the patients presented with symptoms of nasal obstruction and sinusitis. Other signs and symptoms included nasal drainage, meningitis, and visual loss [3]. There were some asymptomatic cases.

The histology of nasal glial heterotopia is characterized by mature glial cells (astrocytes and oligodendrocytes) in stromal connective tissue, covered by the respiratory epithelium [9]. The difference between nasal glial heterotopia and encephalocele has not been clarified pathologically yet [14]. Encephalocele is protrusion of the brain substance connected to the rest of the brain by a pedicle with an associated osseous defect [15]. Nasal glial heterotopia is thought to represent basal or frontal encephalocele that has lost its intracranial meningeal connection [3]. Therefore, nasal glial heterotopia has no connection with the subarachnoid space or central nervous system. Differentiating these conditions is based on clinical and radiological features. The Furstenberg test examines whether the mass becomes enlarged when the ipsilateral jugular vein is compressed or not. This test is typically positive when encephalocele is involved [9]. CT is useful for the visualization of bony defects in the anterior skull base, whereas MRI provides complementary information regarding the fluid or soft tissue characteristics of the mass [7]. Commonly, MRI T2-weighted imaging can reveal hyperintensity related to gliosis and isointensity related to normal respiratory mucosa and indicates the absence of a CSF space or subarachnoid space connection with the lesion. The best imaging tool is multiplanar sagittal MRI which can delineate pedicle of fibrous tissue between the intranasal heterotopia and the intracranial cavity.

Unilateral nasal polyps and tumors in adults are very common conditions encountered by the otolaryngologist. Differential diagnosis of these lesions is made by determining the form, shape, color, and hardness of the mass. The underlying etiology may be inflammation, chronic fungal sinusitis, benign neoplasm, inverted papilloma, or malignant tumor. A radiological evaluation is mandatory to assess the site, extent, and enhancement effect of the lesion. The presence or absence of bony erosion should also be examined. In particular, when intraglial heterotopia is surrounded by respiratory mucosa, it is difficult to discriminate it from such masses as polyps.

In the present case, nasal obstruction had progressed for one year. The CT showed that an isodense shadow occupied the right nasal cavity, maxillary, anterior ethmoid, and frontal sinuses, with no bony erosion, although a small defect in the cribriform plate was noted. The MRI showed that the tumor was solid and had no connection with the meninges or cerebral tissue. We suspected that the tumor was benign or of low grade malignancy. We did not perform biopsy, since the patient had chronic heart failure and cerebral infarction treated with aspirin and warfarin. We decided to perform total endoscopic resection with the additional treatment of low dose heparin. We used low dose heparin (15,000 U/day) for four days before the surgery instead of aspirin and warfarin.

During the operation, the mass was found to arise from the frontal sinus. Defects in the bones surrounding the sinuses or CSF leakage were not observed. However, the patient developed convulsions and frontal lobe cerebral hemorrhage after surgery.

Intranasal glial heterotopia has a firm attachment to the nasal vault at the middle or superior turbinate [12]. An intracranial connection consisting of fibrous stalk and occasionally communication through a cranial bone defect located on the cribriform plate has been reported in 15% to 20% of cases [4, 10]. Our patient had a defect in the cribriform plate and bleeding tendency. We suspected that the force of the surgical procedure was transmitted intracranially, causing the frontal lobe cerebral hemorrhage, near the bone defect. He recovered from the cerebral hemorrhage without dyskinesia. For 2 years after surgery, he has been free from nasal complaints or signs of recurrence. The risk of recurrence following inadequate primary excision is between 4% and 10% [13].

Diagnosis of intranasal glial heterotopia in adults can be made histologically. The presence of a single, unilateral, nontranslucent, noncompressible mass may arouse suspicion of intranasal glial heterotopia or encephalocele. In either case, one should avoid performing biopsy or surgery before a possible intracranial connection is evaluated by CT and MRI.

## 4. Summary

- Nasal glial heterotopia is a rare congenital lesion that is frequently diagnosed in newborns or infants, although this case is found in adults.
- Total endoscopic resection was performed without the CSF leakage; however, the patient developed frontal lobe cerebral hemorrhage after surgery.
- We suspected that the force of the surgical procedure was transmitted intracranially, through the bone defect of cribriform plate.

## References

[1] Fletcher CD, Carpenter G, McKee PH (1986). Nasal glioma. A rarity. *The American Journal of Dermatopathology*.

[2] Younus M, Coode PE (1986). Nasal glioma and encephalocele: two separate entities. Report of two cases. *Journal of Neurosurgery*.

[3] Penner CR, Thompson LDR (2003). Nasal glial heterotopia: a clinicopathologic and immunophenotypic analysis of 10 cases with a review of the literature. *Annals of Diagnostic Pathology*.

[4] Hughes GB, Sharpino G, Hunt W, Tucker HM (1980). Management of the congenital midline nasal mass: a review. *Head and Neck Surgery*.

[5] Gorenstein A, Kern EB, Facer GW, Laws ER (1980). Nasal gliomas. *Archives of Otolaryngology*.

[6] Rouev P, Dimov P, Shomov G (2001). A case of nasal glioma in a new-born infant. *International Journal of Pediatric Otorhinolaryngology*.

[7] Majithia A, Liyanage SH, Hewitt R, Grant WE (2010). Adult nasal glioma presenting with visual loss. *Journal of Laryngology and Otology*.

[8] Pasquini E, Farneti G, Giausa G, Biavati M (1998). A rare case of nasal glioma in adult age. *Otolaryngology—Head and Neck Surgery*.

[9] Altissimi G, Ascani S, Falcetti S, Cazzato C, Bravi I (2009). Central nervous system tissue heterotopia of the nose: case report and review of the literature. *Acta Otorhinolaryngologica Italica*.

[10] Nayak DR, Pujary K, Valiathan M, Parul P, Kamat A (2007). Sphenochoanal polyp with heterotopic glial tissue. *Journal of Laryngology and Otology*.

[11] Herley E (1991). Pediatric congenital masses. *Ear, Nose & Throat*.

[12] Morgan DW, Evans JNG (1990). Developmental nasal anomalies. *Journal of Laryngology and Otology*.

[13] Katz A, Lewis JS (1971). Nasal gliomas. *Archives of Otolaryngology*.

[14] Rahbar R, Resto VA, Robson CD (2003). Nasal glioma and encephalocele: diagnosis and management. *Laryngoscope*.

[15] Yeoh GPS, Bale PM, de Silva M (1989). Nasal cerebral heterotopia: the so-called nasal glioma or sequestered encephalocele and its variants. *Pediatric Pathology*.

